# A Mendelian randomization study for drug repurposing reveals bezafibrate and fenofibric acid as potential osteoporosis treatments

**DOI:** 10.3389/fphar.2023.1211302

**Published:** 2023-07-20

**Authors:** Xiao-Hua Li, Wei-Wei Pang, Yue Zhang, Dan-Yang Liu, Qiao-Rong Yi, Ning Wang, Fu-Rong Zhang, Yun Deng, Xiang-Ding Chen, Jonathan Greenbaum, Hong-Mei Xiao, Hong-Wen Deng, Li-Jun Tan

**Affiliations:** ^1^ Laboratory of Molecular and Statistical Genetics, College of Life Sciences, Hunan Normal University, Changsha, Hunan, China; ^2^ School of Physical Education, Hunan University of Arts and Science, Changde, Hunan, China; ^3^ Zebrafish Genetics Laboratory, College of Life Sciences, Hunan Normal University, Changsha, China; ^4^ Tulane Center of Biomedical Informatics and Genomics, Deming Department of Medicine, Tulane University School of Medicine, New Orleans, LA, United States; ^5^ Center for System Biology, Data Sciences, and Reproductive Health, School of Basic Medical Science, Central South University, Changsha, China

**Keywords:** osteoporosis, Mendelian randomization, drug repurposing, lipid-lowering drugs, bezafibrate and fenofibric acid

## Abstract

**Background:** Lipid pathways have been implicated in the pathogenesis of osteoporosis (OP). Lipid-lowering drugs may be used to prevent and treat OP. However, the causal interpretation of results from traditional observational designs is controversial by confounding. We aimed to investigate the causal association between genetically proxied lipid-lowering drugs and OP risk.

**Methods:** We conducted two-step Mendelian randomization (MR) analyses to investigate the causal association of genetically proxied lipid-lowering drugs on the risk of OP. The first step MR was used to estimate the associations of drug target genes expression with low-density lipoprotein cholesterol (LDL-C) levels. The significant SNPs in the first step MR were used as instrumental variables in the second step MR to estimate the associations of LDL-C levels with forearm bone mineral density (FA-BMD), femoral neck BMD (FN-BMD), lumbar spine BMD (LS-BMD) and fracture. The significant lipid-lowering drugs after MR analyses were further evaluated for their effects on bone mineralization using a dexamethasone-induced OP zebrafish model.

**Results:** The first step MR analysis found that the higher expression of four genes (*HMGCR*, *NPC1L1*, *PCSK9* and *PPARG*) was significantly associated with a lower LDL-C level. The genetically decreased LDL-C level mediated by the *PPARG* was significantly associated with increased FN-BMD (BETA = −1.38, *p* = 0.001) and LS-BMD (BETA = −2.07, *p* = 3.35 × 10^−5^) and was marginally significantly associated with FA-BMD (BETA = −2.36, *p* = 0.008) and reduced fracture risk (OR = 3.47, *p* = 0.008). Bezafibrate (BZF) and Fenofibric acid (FBA) act as *PPARG* agonists. Therefore genetically proxied BZF and FBA had significant protective effects on OP. The dexamethasone-induced OP zebrafish treated with BZF and FBA showed increased bone mineralization area and integrated optical density (IOD) with alizarin red staining.

**Conclusion:** The present study provided evidence that BZF and FBA can increase BMD, suggesting their potential effects in preventing and treating OP. These findings potentially pave the way for future studies that may allow personalized selection of lipid-lowering drugs for those at risk of OP.

## 1 Introduction

Osteoporosis (OP) is a systemic skeletal disease that is characterized by a deterioration of bone quality and loss of bone density, causing compromised bone strength and increased risk of low trauma fractures (Tarantino et al., 2017). To date, OP has attracted extensive attention worldwide (O’Neill et al., 1996), and various pharmacological treatments have been developed, such as bisphosphonates, denosumab, odanacatib, and romosozumab, which act by reducing the rate of bone resorption by osteoclasts (Reid, 2011; Dahiya et al., 2015). On the other hand, teriparatide is an anabolic intervention that stimulates bone formation by osteoblasts. The current treatments are not satisfactory as disease cures and can also have major side effects. For example, although most studies have shown that teriparatide increases bone mineral density (BMD) and prevents fractures (Neer et al., 2001; Miyauchi et al., 2010), this treatment has been associated with an unusual incidence of hypercalcemia (Hajime et al., 2014). Therefore, it is crucial to find new medicines promoting bone formation.

BMD is the most powerful known risk factor for predicting fracture risk, as well as the most commonly utilized phenotype for clinically diagnosing OP (Rachner et al., 2011). The relationship of BMD with serum lipids has been extensively researched. In general, epidemiological findings regarding the association between lipid profile and BMD are inconsistent (Li et al., 2020). Although some studies have shown no association between dyslipidemia and BMD, others have reported either a negative or a positive effect for different lipid factors (Poli et al., 2003; Adami et al., 2004; Orozco, 2004; Cui et al., 2005). Several, though not all, evidence have shown an inverse association of BMD with total cholesterol (TC) and LDL-C (Mandal, 2015). It has been suggested that increased cholesterol inhibits osteoblast differentiation, prevents bone formation, and may also enhance osteoclastogenesis (Che et al., 2022). Additionally, different effects of serum cholesterol on BMD at various skeletal sites have been suggested (Cui et al., 2005), which may partially explain the inconsistency among studies.

Statins are one of the most commonly prescribed medications worldwide for lowering circulating LDL-C levels (Sirtori, 2014). Statins have been reported to influence bone by regulating the proliferation and differentiation, of osteoblasts, as well as by decreasing osteoclastogenesis (Oryan et al., 2015). Animal experiments suggested that simvastatin can improve related inflammatory cytokines and serum biochemical indicators in OP rats, confirming that simvastatin can promote the differentiation of mesenchymal stem cells (MSCs) into osteoblasts in the OP rat model through the BMP-2/Smads signaling pathway (Feng et al., 2020). In addition to the beneficial effects of commonly used statin lipid-lowering drugs on bone metabolism, Wang et al. (2017) found that the DPP-4 inhibitor sitagliptin can alleviate the loss of bone mass in ovariectomized mice by inhibiting RANKL-induced osteoclast differentiation. Huang et al. (2019) found that 20(S)-hydroxycholesterol and simvastatin synergistically enhance osteogenic differentiation of MSCs by initiating Raf/MEK/ERK signaling. Currently, there is no research to definitively prove the causal relationship between statin therapy and OP. A case-control study by Cheng et al. (2019) reported that the current use of statins seemed to have a protective effect against hip fractures in older people. In contrast, Leutner et al. reported that a high dose of statin use was associated with an increased risk of OP. Therefore, the role of lipid-lowering drugs in the prevention of OP requires further investigation.

Drug repurposing refers to the identification of novel applications for existing drugs. Compared with traditional drug development, drug repurposing recommends potential therapeutic drugs in a faster and more cost-effective way (Calcoen et al., 2015). Mendelian randomization (MR) is a method designed for causal inference using germline genetic variants, which are randomly allocated at conception, to construct instrumental variables (Smith and Ebrahim, 2003). Thus, MR can minimize the problems of confounding bias and reverse causality, which is common in traditional epidemiological studies. MR analysis has been proposed as an efficient method to infer the influence of drugs targeting protein encoding genes on disease risk (Walker et al., 2017). MR analysis produced instruments applying genetic variants that were located within or near drug target genes. The expression and function of drug targets may be affected by genetic variants, and the effects of drugs can be predicted by genetic variants in their protein target genes (Chauquet et al., 2021). Walker et al. (2020) employed this approach and successfully repurposed antihypertensive drugs to reduce Alzheimer’s disease risk. Using a similar study design, Liu et al. (2021) found that LDL-C lowering drugs were associated with a higher risk of renal cell carcinoma in men.

In this study, we investigated the causal association between genetically proxied drug targets of lipid-lowering drugs and OP by using a two-sample MR analysis. We employed publicly available expression quantitative trait locus (eQTL) data to identify suitable genetic instruments for lipid-lowering drug target gene expression. We performed MR analyses to estimate the causal effects of variants in genes encoding lipid-lowering drug targets on the risk of OP using summary data from the genome-wide association studies (GWAS). The identified interesting drugs were further evaluated for their potential utility to prevent OP in a dexamethasone-induced OP zebrafish model.

## 2 Methods

### 2.1 Study design

**FIGURE 1:**
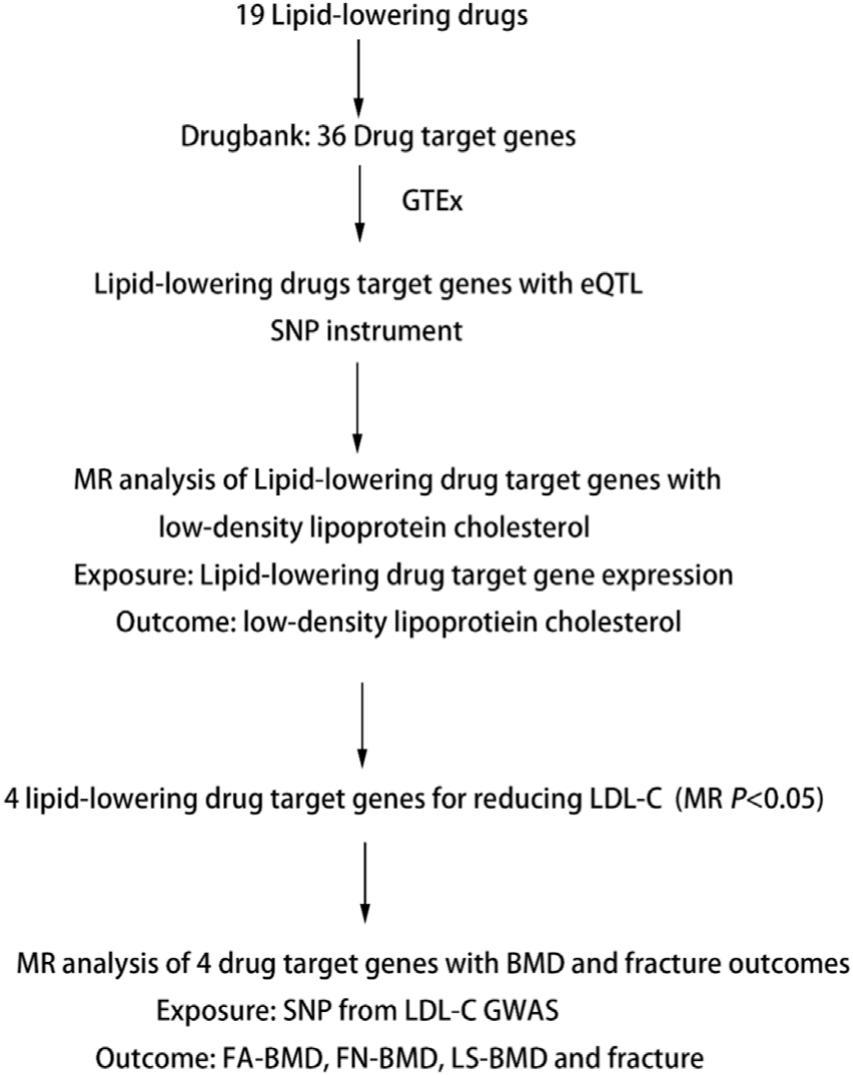
Summary of Study Design. Note: eQTL, expression quantitative trait loci; SNP, single nucleotide polymorphism; MR, Mendelian randomization; BMD, bone mineral density; FA-BMD, forearm bone mineral density; FN-BMD, femoral neck bone mineral density; LS-BMD, lumbar spine bone mineral density.

We performed two-step MR analyses to investigate the association of genetically proxied lipid-lowering drug targets on the risk of OP. We performed first step MR to estimate the causal effects of drug target genes expression from eQTL studies, in which the most significant cis-eQTL SNP was selected as a genetic instrument for the target gene expression of each approved lipid-lowering drug, with LDL-C levels using summary data from GWAS. The significant SNPs in the first step MR were used in the second step MR to estimate the causal effects of LDL-C levels with forearm BMD (FA-BMD), femoral neck BMD (FN-BMD), lumbar spine BMD (LS-BMD), and fracture (Table 1). Details of the study design are shown in Figure 1.

**TABLE 1:** Lipid-lowering drug classes, substances and targets with their DrugBank ID.

Drug	Substance	Drugbank_id	Gene
Statins	Atorvastatin	DB01076	*HMGCR; DPP4; AHR; HDAC2; NR1I3*
	Fluvastatin	DB01095	*HMGCR; HDAC2*
	Pravastatin sodium	DB00175	*HMGCR; HDAC2*
	Rosuvastatin	DB01098	*HMGCR; ITGAL*
	Simvastatin	DB00641	*HMGCR; ITGAL; HDAC2*
	Lovastatin	DB00227	*HMGCR; ITGAL*
	Alirocumab	DB09302	*PCSK9*
	Fish oil	DB13961	*DGAT2; PTGS2; FFAR4*
	Pitavastatin	DB08860	*HMGCR; ITGAL*
Ezetimibe	Ezetimibe	DB00973	*NPC1L1; SOAT1; ANPEP*
Fibrates	Bezafibrate	DB01393	*PPARA; PPARD; PPARG; NR1I2; RXRA; RXRB; RXRG*
	Ciprofibrate	DB09064	*PPARA*
	Fenofibrate	DB01039	*PPARA; MMP25; NR1I2*
	Gemfibrozil	DB01241	*PPARA; SLCO1B1; SLC22A8; SLCO2B1; SLCO1B3*
	Fenofibric acid	DB13873	*PPARA; MMP25; PPARG; PPARD; NR1I2*
Nicotinic acid group	Acipimox	DB09055	
	Nicotinic acid	DB00627	*HCAR3; HCAR2; QPRT; NNMT*
Omega-3 fatty acid compounds	Omega-3-acid ethyl esters	DB09539	*SREBF1*
	Omega-3-carboxylic acids	DB09568	*DGAT2; HSD17B10; ECHS1; HADH; ELOVL4; LPL*

### 2.2 Selection of drug target genes

We searched six classes of Lipid-regulating drugs in the British National Formulary including statins, bile acid sequestrants, ezetimibe, fibrates, nicotinic acid group, and Omega-3 fatty acid compounds. Using the drug substance information, we identified the active protein targets and the corresponding genes of lipid-lowering drugs by searching the DrugBank database (https://www.drugbank.ca/; version 5.1.1) (Table 1) (Wishart et al., 2006).

### 2.3 Genetic instruments for first step MR analysis

The summary data for gene expression of lipid-lowering drug target genes in all tissues were selected from Genotype-Tissue Expression (GTEx) Consortium (https://gtexportal.org/) (Battle et al., 2017). We selected cis-eQTL genetic instruments significantly (minor allele frequency (MAF) > 1% and *p* < 1 × 10^−5^) associated with the expression of genes that were minimally correlated (linkage disequilibrium threshold of *r* ^2^ < 0.1 from the 1,000 Genomes Project European data) within 1 Mb on either side of the encoded genes. When multiple eQTL signals were present for a particular candidate gene across different tissues, the most significant (minimal *p*-value) eQTLs were selected. A total of 36 lipid-lowering drug target genes and 4,283 cis-eQTL variants were selected for the subsequent analysis. The description of the number of variants was shown in Supplementary Table S1.

### 2.4 GWAS data source

The Summarized data on LDL-C were obtained from the GWAS of the Global Lipids Genetics Consortium (GLGC) (Willer et al., 2013). GLGC is a meta-analysis to identify genetic variants associated with circulating LDL-C levels. This study included 173,082 subjects and 2,437,752 SNPs, and the meta-analysis was restricted to participants of European ancestry. The fenofibrinic acid (FA), femoral neck (FN), and lumbar spine (LS) are the three most common skeletal sites for measurement of BMD based on dual-energy x-ray absorptiometry (DXA) (Zheng et al., 2015). We obtained publicly available GWAS summary data of FA-BMD (SNP = 9,955,366, *n* = 8,143), FN-BMD (SNP = 10, 586, 900, *n* = 32,735), LS-BMD (SNP = 10, 582, 867, *n* = 28,498) and fracture (SNP = 2,539,801, *n* = 264,973) from Genetic Factors for OP Consortium (GEFOS, http://www.gefos.org/) (Estrada et al., 2012), which is the largest GWAS on DXA-measured BMD and fracture to date.

### 2.5 MR analysis

We performed the first step MR to estimate the causal association of drug target gene expression of lipid-lowering drugs with LDL-C using target genes expression (using the eQTL data) as exposure and LDL-C as the outcome (Bowden and V Holmes, 2019). The inverse variance weighted (IVW) method was used to estimate the causal effect between the exposure and outcome. The SNPs which were associated with LDL-C were used as the instruments in further MR analysis and were shown in Supplementary Table S2. The effect size estimated from MR for the association between gene expression and LDL-C represented the changes in LDL-C per 1-SD increase in gene expression levels (Chen et al., 2022).

We performed the second MR analysis to estimate the causal association of LDL-C lowering by drug target gene expression with outcomes of interest (using GWAS summary data for FA-BMD, FN-BMD, LS-BMD and fracture). The effect size suggested the potential effect and direction of association of LDL-C lowering by drug target gene expression with risk of FA-BMD, FN-BMD, LS-BMD and fracture. IVW method was used, which essentially assumed the intercept was zero and associated a weighted regression of SNP-exposure effects with SNP-outcome effects. SNPs absent in the outcome data were replaced by proxy SNPs in high linkage disequilibrium (*r* ^2^ ≥ 0.8) from the 1,000 Genomes Project European data. The significance threshold was determined using the Bonferroni method to adjust for multiple hypothesis testing. *p* < 1.16 × 10^−5^ (0.05/4,283) in the first MR analysis and *p* < 0.0056 (0.05/9) in the second MR analysis were considered to be significant.

### 2.6 Gene set enrichment analysis (GSEA)

To explore the potential biological function pathways for lipid-lowering drugs involved in OP, we performed GSEA to analyze drug and disease-related biological pathways by examining the distribution of disease genes in drug response gene expression profiles. GSEA was utilized to determine whether a predefined set of genes (usually from functional annotations or results from previous experiments) showed statistically significant enrichment in a collected gene list (usually from gene expression profiles). This analysis could identify whether drug treatment affected the expression of disease-related pathways in this study (Subramanian et al., 2005). We first collected 213 genes from the human disease database MalaCards (Rappaport et al., 2017), 295 genes from GAD (Genetic Association Database) (http://geneticassociationdb.nih.gov), 88 genes from the GWAS catalogue (https://www.ebi.ac.uk/gwas/), 39 genes from OMIM (Online Mendelian Inheritance in Man) (https://www.omim.org/) by searching public databases with “osteoporosis.” After deleting the overlapped genes, a total of 528 OP genes were selected. Then, we acquired the lipid-lowering drug bezafibrate (BZF) and fenofibrate gene expression profiles from LINCS (Library of Integrated Network-based Cellular Signatures), which provides transcriptional responses of human cells to chemical and genetic perturbation (Zhao et al., 2021). Since the Fenofibric Acid (FBA) gene expression profile was not available in LINCS, we used the fenofibrate gene expression profile for GSEA analysis. The myoblast cell line was selected due to the known interactions of skeletal muscle with bone (Li et al., 2019). GSEA was conducted using the Cluster Profiler package of R software, and *P*. adjust <0.05 were considered significantly enriched.

### 2.7 Validation of effects of candidate drugs on zebrafish

To further evaluate the effect of candidate drugs with significant causal effects in MR on bone mineralization, we established dexamethasone (Dex)-induced zebrafish OP model and exposed zebrafish larvae to different concentrations of each drug candidate. Zebrafish is a powerful animal model to study bone development given the high degree of similarity of both bone architecture and genetics with those seen in humans (Pasqualetti et al., 2015). Zebrafish has transparent body for easy viewing of the skeleton and has a short reproductive cycle. Dexamethasone-induced zebrafish is a commonly used model in evaluating drug efficacy in drug discovery, which exhibits an osteoporotic phenotype after treatment with and dexamethasone (Saito et al., 2020). Dex-induced zebrafish is a commonly used glucocorticoid-induced OP model for evaluating drug efficacy in drug discovery, in which drug treatments that alleviate Dex-induced OP in zebrafish are thought to activate bone remodeling and be effective for the treatment of OP (Saito et al., 2020).

FBA, Dimethylsulfoxide (DMSO) and Dexamethasone (Dex) were provided by the Abmole Company (Houston, United States). Wild-type TU (Tuebingen) Zebrafish, provided by the Department of Genetics and Development Biology, College of Life Sciences in Hunan Normal University, were bred in natural pairs in 28°C water for fish culture. Zebrafish larvae of 3 days post fertilization (dpf) were placed in 6-well plates and randomly divided into four groups: 0.04% DMSO (control) group, 10 µm Dex (model) group, 10 µm Dex + BZF (10,1 μg/mL) group, 10 µm Dex + FBA (10,1 μg/mL) group from 3 dpf to 9 dpf (Yang et al., 2019). Zebrafish larvae were cultured in a 28°C constant temperature incubator, aspirated half of the test solution every 24 h, added the same drug-containing culture water to keep the original volume unchanged from 3 dpf to 9 dpf. The culture water was changed in each 12 h at 0 dpf to 3 dpf. Because the Yolk sac of wild type Zebrafish can provide nutrients for embryo growth and development within 9 dpf, it does not need to be fed during this period.

Zebrafish larvae were collected and fixed in 4% paraformaldehyde for 1 hour at 9 dpf. After washing with 100 mM Tris/10 mM MgCl_2_, Zebrafish larvae was bleached with 3% H_2_O_2_/0.5% KOH until the eyes of Zebrafish become transparent. Next, these fish larvae were rinsed with 25% glycerol/0.1% KOH until no bubble generation. After removing the rinsing solution, the larvae were stained with 0.01% Alizarin red solution for 1 hour, with 50% glycerol/0.1% KOH to stain formed bone for 10 min and subsequently washed twice with 25% glycerol/0.1% KOH.

Finally, the photoes were obtained digitally using a fluorescent stereomicroscope. We used Image pro plus 6.0 (America Media cybernetics) to select the alizarin red stained area, and calculated its area and integrated optical density (IOD). SPSS 12.0 software was used to calculate the means and standard deviation (SD) of different groups, and *t*-test was used to test for a significant difference between different groups. The analyses are corrected for multiple testing by applying the Bonferroni correction. We divided *p* = 0.05 by the number of tests (10) to get the Bonferroni critical value, so a test would have to have *p* < 0.005 to be significant. The sample size for each treatment was eight embryos per well, with three technical replicates.

## 3 Results

### 3.1 MR estimates

In the Drugbank database, we identified a total of 36 genes whose encoded protein activity has been experimentally shown to be modified by one or more lipid-lowering drugs (Table 1). In the GTEx database, we extracted the cis-eQTL SNPs associated with the expression of the 36 drug target genes. The first MR analysis showed that the higher expression of four genes (*HMGCR*, *NPC1L1*, *PCSK9* and *PPARG*) was associated with the lower LDL-C level (Table 2; Supplementary Table S2).

**TABLE 2:** MR results for the association of the genes expression of lipid-lowering drugs targets with genetically inferred LDL-C.

Gene	SNP	Beta	95% CI		*p*-value
			Lower	Upper	
*PPARG*	rs310749	−0.183	−0.259	−0.107	1.97E-06
*HMGCR*	rs3846662	−0.384	−0.425	−0.343	4.26E-74
*PCSK9*	rs11583974	−0.248	−0.336	−0.160	3.36E-08
*NPC1L1*	rs41279633	−0.109	−0.137	−0.081	8.41E-14

Note: MR, Mendelian randomization; SNP, single nucleotide polymorphism; LDL-C, low density lipoprotein cholesterol.

**FIGURE 2:**
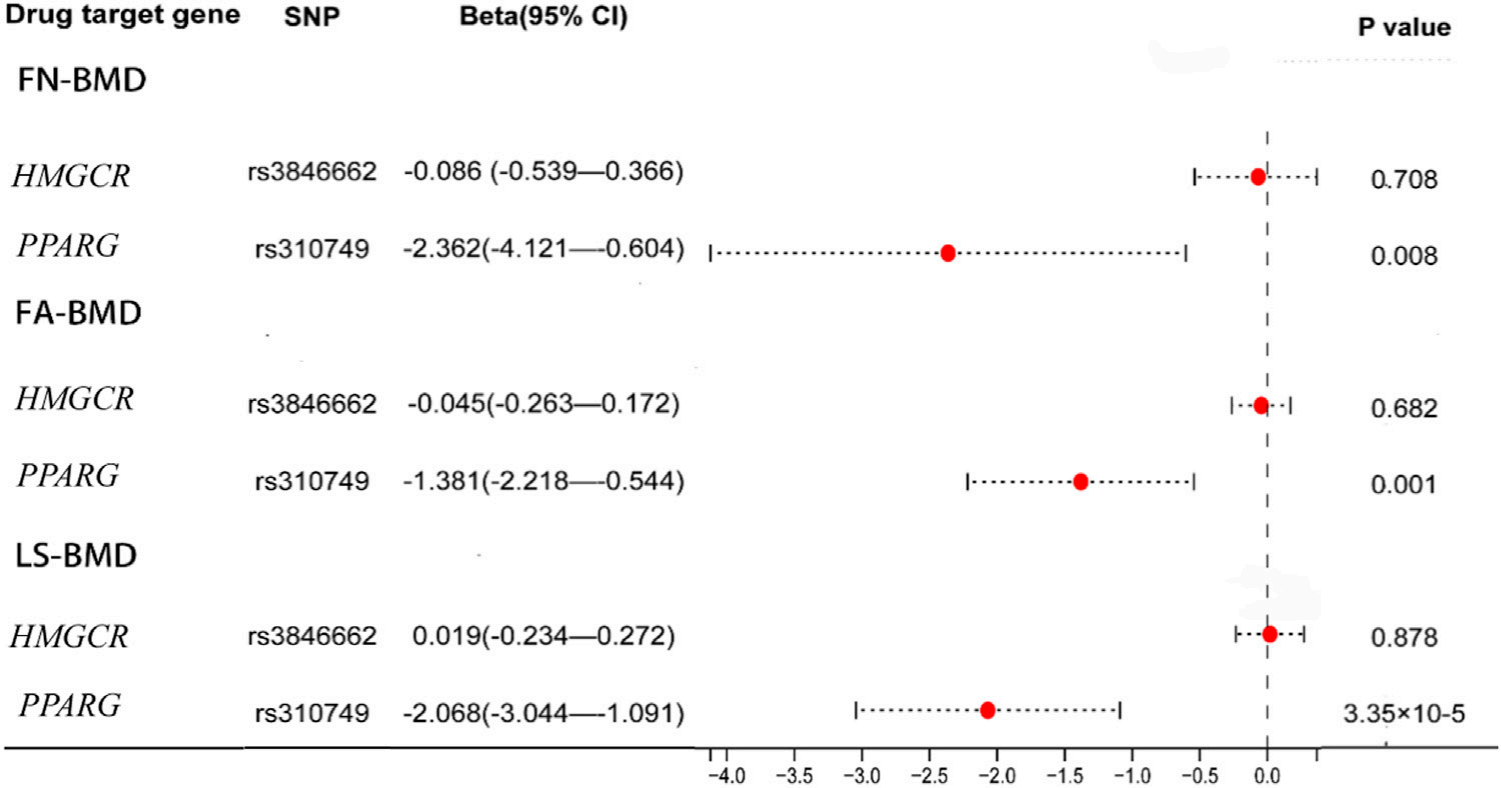
Association of the genetically inferred LDL-C level with FA-BMD, FN-BMD and LS-BMD. Note: Mendelian randomization estimates for the effects of exposure to drug target modulation on Forearm bone mineral density (FA-BMD), Femoral neck bone mineral density (FN-BMD) and Lumbar spine bone mineral density (LS-BMD). SNP, single nucleotide polymorphism; CI, confidence interval.

**FIGURE 3:**
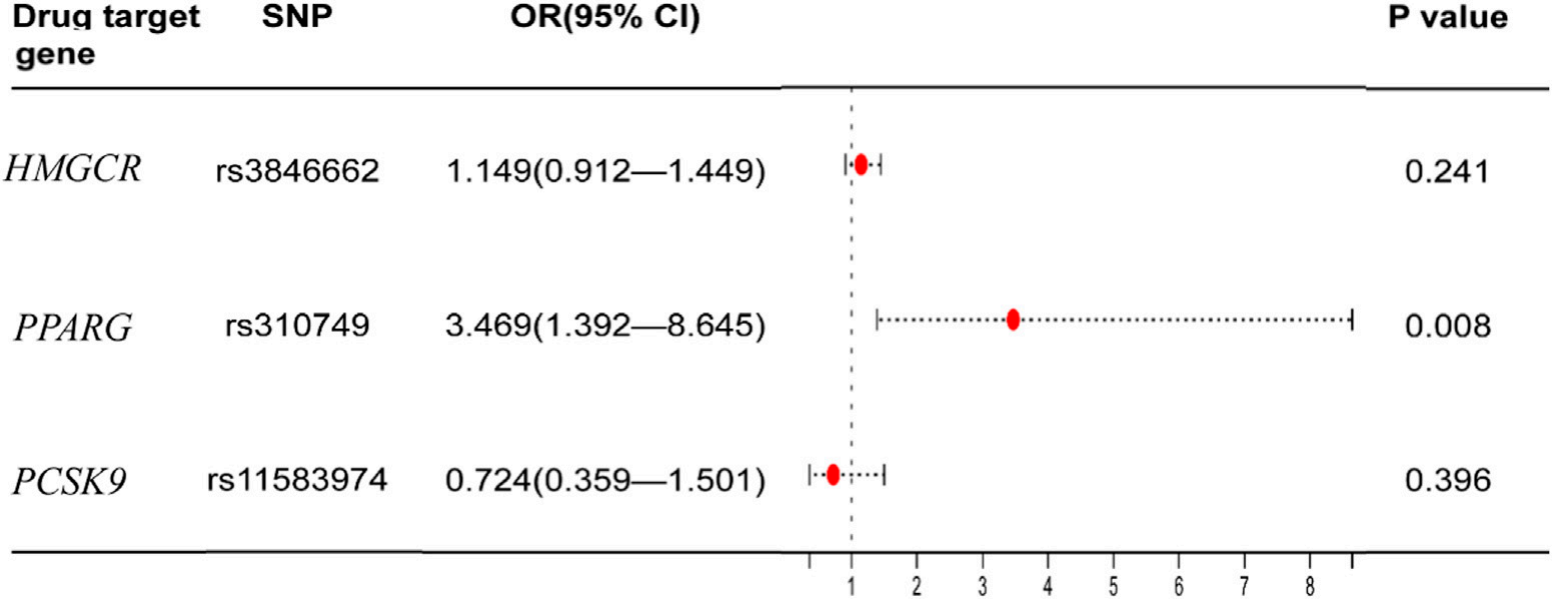
Association of the genetically inferred LDL-C level with fracture. Note: Mendelian randomization estimates for the effects of exposure to drug target modulation on fracture. SNP, single nucleotide polymorphism; CI, confidence interval; OR, odds ratio.

The significant SNPs in the first MR analysis were extracted in the GWAS data of LDL-C as the instrumental variables for the second MR analysis. Results of genetically inferred LDL-C on BMD suggested that genetically decreased LDL-C level mediated by the *PPARG* gene (i.e., mimicking the effect of BZF and FBA) was significantly associated with increased genetically inferred FN-BMD (BETA = −1.38, 95% CI: −2.22 to −0.54, *p* = 0.001) and LS-BMD (BETA = −2.07, 95% CI: −3.04 to −1.09, *p* = 3.35 × 10^−5^) (Table 3; Figure 2). Genetically decreased LDL-C level mediated by the *PPARG* gene showed marginally significant association with increased FA-BMD (BETA = −2.36, *p* = 0.008) and reduced fracture risk (OR = 3.47, *p* = 0.008) (Table 4; Figure 3). BZF and FBA act as *PPARG* agonists. Therefore genetically proxied BZF and FBA had significant protective effects on OP. In contrast, we found that genetically reducing the LDL-C level mediated by *HMGCR* had no significant effect on FA-BMD (*p* = 0.71), FN-BMD (*p* = 0.682), LS-BMD (*p* = 0.878), and fracture (*p* = 0.24) (Tables 3, 4 and Figures 2, 3).We also observed that genetically reducing the LDL-C level mediated by *P*CS*K9* had no significant effect on fracture (*p* = 0.40) (Table 4; Figure 3). Due to the absence of identical SNPs and proxy SNPs in BMD and fracture GWAS data for rs41279633 of the *NPC1L1* gene and rs11583974 of the *PCSK9* gene, *NPC1L1* and *PCSK9* genes with BMD and *NPC1L1* with fracture were excluded in the second MR analysis.

**TABLE 3:** MR Results for the association of genetically inferred LDL-C with FA-BMD, FN-BMD, LS-BMD.

	Gene	SNP	Beta	95% CI		*p*-value	
				Lower	Upper		
FA-BMD	*PPARG*	rs310749	−2.362	−4.121	−0.604	0.008	
	*HMGCR*	rs3846662	−0.086	−0.539	0.366	0.708	
FN-BMD	*PPARG*	rs310749	−1.381	−2.218	−0.544	0.001	
	*HMGCR*	rs3846662	−0.045	−0.263	0.172	0.682	
LS-BMD	*PPARG*	rs310749	−2.068	−3.044	−1.091	3.35E-5	
	*HMGCR*	rs3846662	0.019	−0.234	0.272	0.878	

Note: MR, Mendelian randomization; SNP, single nucleotide polymorphism; FA-BMD, forearm bone mineral density; FN-BMD, femoral neck bone mineral density; LS-BMD, lumbar spine bone mineral density; CI: confidence interval.

**TABLE 4:** MR results for the association of LDL-C with fracture.

	Gene	SNP	OR	95% CI		*p*-value
				Lower	Upper	
	*PPARG*	rs310749	3.469	1.392	8.645	0.008
Fracture	*HMGCR*	rs3846662	1.149	0.912	1.449	0.241
	*PCSK9*	rs11583974	0.734	0.359	1.501	0.396

Note: MR, Mendelian randomization; SNP, single nucleotide polymorphism; CI: confidence interval; OR, odds ratio.

### 3.2 GSEA

**FIGURE 4:**
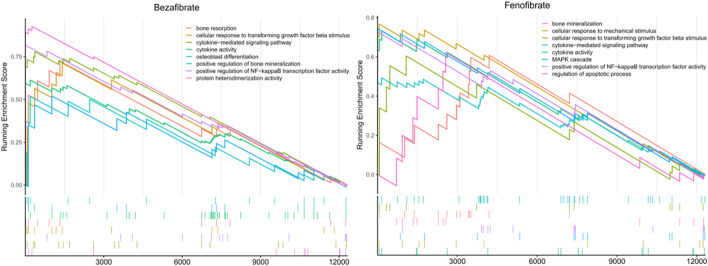
The potential biological function pathways by gene set enrichment analysis using the BZF and fenofibrate -treatment gene expression profile in LINCS database.

In the GSEA analysis of BZF and fenofibrate-treatment gene expression profile in OP-related genes, a total of 83 and 25 biological pathways with *P*. adjust <0.05 were significantly enriched in BZF and fenofibrate-treatment gene expression profile, respectively (Figure 4). The enriched biological pathways included osteoblast differentiation, positive regulation of bone mineralization, positive regulation of NF-kappaB, bone resorption, which were related to bone metabolism. We speculated that the mechanism of BZF in bone metabolism may act through up-regulating these signaling pathways (Zhong et al., 2011a) (Figure 4).

### 3.3 Effects of BZF and FBA on bone mineralization

**FIGURE 5:**
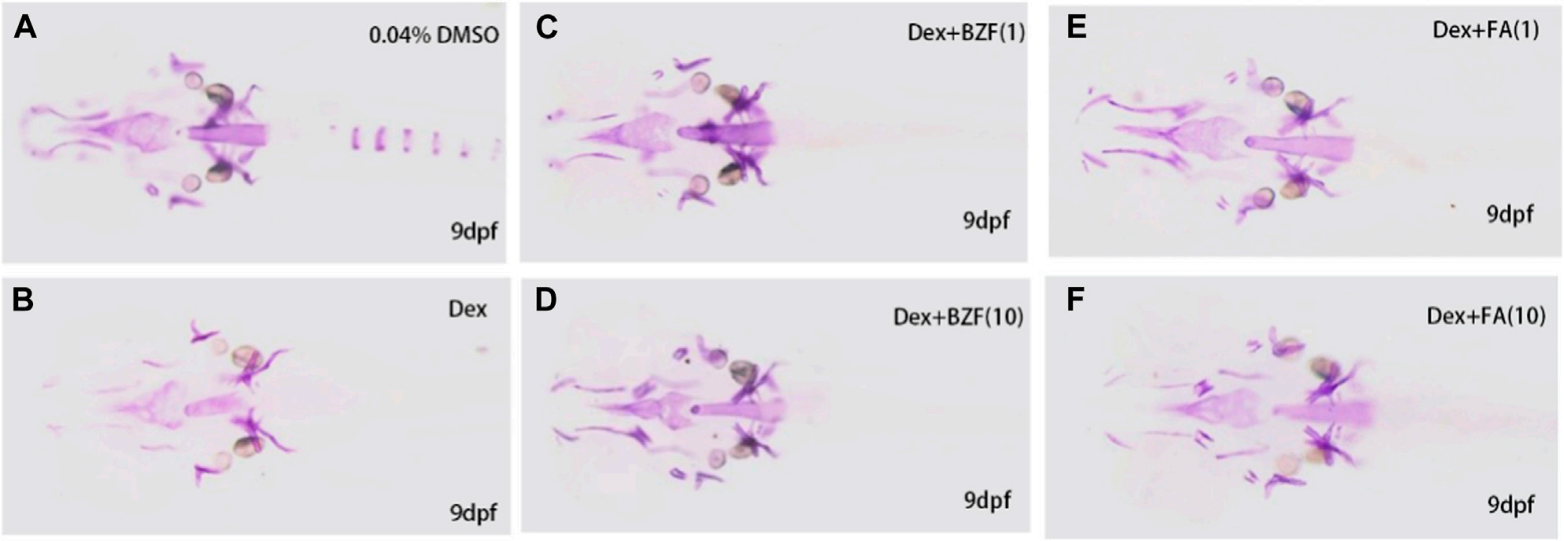
Effect of BZF and FBA on osteoporosis induced by dexamethasone in zebrafish. Note: Zebrafish Alizarin Red Staining **(A–F)**, **(A)** 0.04% Dimethylsulfoxide; **(B)** 10 µm Dexamethasone, **(C, D)** 10 µm Dexamethasone + BZF (1,10 μg/mL), **(E, F)** 10 µm Dexamethasone + FBA (1,10 μg/mL).

**FIGURE 6:**
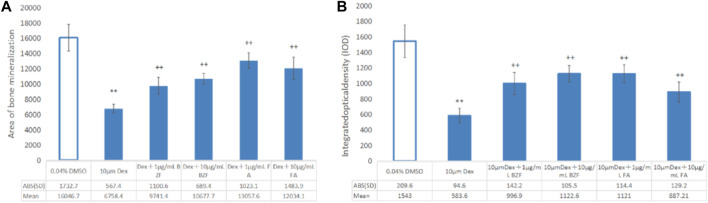
Effect of BZF and FBA on osteoporosis induced by dexamethasone in zebrafish. **(A)** Bone mineral area; **(B)** IOD bar chart; **Compared with the 0.04% DMSO group, *p* < 0.001; ++ Compared with the 10 µm Dexamethasone group, *p* < 0.001. ABS (SD): the absolute value of “±SD”.

Compared with the control group, the bone mineralized area and IOD of the Dex group were significantly decreased (*p* < 0.01), indicating that dexamethasone successfully induced OP in zebrafish. Compared with the Dex group, the bone mineralization area and IOD of the Dex + BZF group and Dex + FBA group increased significantly (*p* < 0.01), indicating that 10 μg/mL and 1 μg/mL BZF and FBA increased the amount of bone mineralization in zebrafish (Figures 5, 6). Compared with the control group (DMSO), the *PPARG* gene expression level in adipose tissue was upregulated by BZF-treatment group in LINCS database (Supplementary Figure S1).

## 4 Discussion

The MR analyses suggested that genetically proxied effects of the lipid-lowering drugs BZF and FBA increased BMD and reduced fracture risk by reducing genetically inferred LDL-C. The zebrafish alizarin red staining experiments also confirmed that BZF and FBA can lower the risk of OP.

BZF and FBA are peroxisome proliferators-activated receptors (PPARs)-alpha agonists, which are a class of medications frequently used to treat hypertriglyceridemia and hypercholesterolemia (Honda et al., 2022). FBA has excellent efficacy on dyslipidemia by monotherapy or in combination with low- and moderate-dose statins (Alagona, 2010). The PPARα agonist fenofibrate was shown to improve BMD in both sham and ovariectomized rats (Mosti et al., 2016). PPAR activities are functionally involved in modulating the interaction between the BMP and TNF-α receptor signaling pathways that are crucial for bone metabolism (Takano et al., 2012). Our GSEA analysis showed osteoblast differentiation, positive regulation of bone mineralization, positive regulation of NF-kappaB, bone resorption were enriched in BZF and fenofibrate-treatment gene expression profile, suggesting that BZF and fenofibrate may play a role in bone metabolism through activating these signaling pathways (Zhong et al., 2011a; Han et al., 2019). In particular, BZF has been demonstrated to increase the number of osteoblastic colonies produced from rat bone marrow stromal cells (BMSCs) (Zhong et al., 2011b). Other *in vivo* and *in vitro* studies also showed that BZF increased osteoblast differentiation and induced periosteal bone formation in rats (Still et al., 2008; Han et al., 2019). Kim et al. (2019) revealed that fenofibrate enhanced the osteoblast differentiation of C3H10T1/2 and MC3T3-E1 cells and showed a stimulatory effect on osteoblast differentiation via the elevation of PPARα levels and the PPARα-mediated BMP2 expression.

Our results indicated that BZF and FBA can increase bone density through LDL-C lowering effect. Several recent MR analyses have similarly suggested a negative causal effect of LDL-C level on BMD. A recent meta-analysis of 10 studies found that LDL-C levels were higher in patients with OP compared with controls (Zheng et al., 2020). An epidemiological study confirmed the association between BMD and LDL-C in two independent cohorts and provided reliable evidence that a reduction in blood LDL-C level was causally associated with increased FN-BMD, LS-BMD, FA-BMD (Li et al., 2020). MR analyses using five SNPs in *HMGCR* gene and LDL-C-related variants also supported the causal effect of LDL-C on BMD (Zheng et al., 2020). Taken together, these observations suggest that gains in BMD after BZF and FBA are at least partly attributable to a causal effect of lowering LDL-C.

However, despite the novelty of this drug repurposing analysis in the bone field, there are several limitations. First, selection bias due to the small sample size of the eQTL data in GTEx cannot be excluded. Future studies with a larger sample size and novel statistical analysis approaches (such as inverse probability weighting method) might produce more robust effect estimates. Second, drug-target MR analysis was devised to reflect the effect of lipid-lowering agents’ life-long modulation to change LDL-C levels on the disease. MR estimations were not able to imply the outcome of short-term administration of lipid-modifying agents. Variables such as drug dose, period of exposure, inter-individual variation in drug metabolism, power to reach relevant tissue and drug-binding affinity all play a role in modifying toxicity and drug efficacy, making it difficult to extrapolate drug exposure’s real effect from genetic analyses (Walker et al., 2020). Third, this study assumed that alterations in gene expression may mirror modifications in protein levels and/or activity, which may not always be the case (Chauquet et al., 2021). To elucidate the anti-OP mechanism of BZF and FBA, future biological validation experiments that investigate how drugs affect normal bone cells such as osteoblasts and osteoclasts will be critical.

In conclusion, the current study provides evidence that genetically proxied *PPARG* agonists BZF and FBA may be causally associated with increased FN-BMD, LS-BMD and FA-BMD, as well as a lower risk of OP. The dexamethasone-induced OP zebrafish experiments confirmed the protective effects of BZF and FBA on bone mineralization, suggesting their potential effects in preventing and treating OP. These findings potentially pave the way for future studies that may allow personalized selection of lipid-lowering drugs for those at risk of OP.

## Data Availability

The original contributions presented in the study are included in the article/Supplementary Material, further inquiries can be directed to the corresponding author.
