# Supplementary material for: A Mendelian randomization study for drug repurposing reveals bezafibrate and fenofibric acid as potential osteoporosis treatments
Source: Front Pharmacol. 2023 Jul 20;14:1211302. doi: 10.3389/fphar.2023.1211302 (PMC10397407; doi:10.3389/fphar.2023.1211302)
Supplement: Supplementary file 1 [file Table1.docx]

Supplementary Table S1 The number of cis-eQTL variants

| Gene | The number of cis-eQTL variants |
| --- | --- |
| AHR | 49 |
| ANPEP | 183 |
| DGAT2 | 80 |
| DPP4 | 33 |
| ECHS1 | 45 |
| ELOVL4 | 373 |
| FFAR4 | 118 |
| HADH | 153 |
| HCAR2 | 60 |
| HCAR3 | 82 |
| HDAC2 | 13 |
| HDAC3 | 46 |
| HMGCR | 117 |
| HSD17B10 | 76 |
| ITGAL | 16 |
| LPL | 190 |
| MMP25 | 60 |
| NNMT | 49 |
| NPC1L1 | 120 |
| NR1I2 | 54 |
| NR1I3 | 15 |
| PCSK9 | 130 |
| PPARA | 87 |
| PPARD | 126 |
| PPARG | 201 |
| PTGS2 | 12 |
| QPRT | 28 |
| RXRA | 75 |
| RXRB | 184 |
| RXRG | 54 |
| SLCO1B1 | 132 |
| SLCO1B3 | 333 |
| SLCO2B1 | 193 |
| SOAT1 | 587 |
| SREBF1 | 211 |
| Total | 4283 |
