# Supplementary material for: A Mendelian randomization study for drug repurposing reveals bezafibrate and fenofibric acid as potential osteoporosis treatments
Source: Front Pharmacol. 2023 Jul 20;14:1211302. doi: 10.3389/fphar.2023.1211302 (PMC10397407; doi:10.3389/fphar.2023.1211302)
Supplement: Supplementary file 2 [file Table2.docx]

Supplementary Table S2 The information regarding the 4 significant SNPs in MR analysis

| **SNP** | **rs310749** | **rs3846662** | **rs11583974** | **rs41279633** |
| --- | --- | --- | --- | --- |
| **Gene** | *PPARG* | *HMGCR* | *PCSK9* | *NPC1L1* |
| **Lipid-lowering drugs** | bezafibrate,  fenofibric acid | atorvastatin, fluvastatin, pravastatin sodium, rosuvastatin, simvastatin, lovastatin | alirocumab | ezetimibe |
| **Tissue in eQTL data from GTEx** | Nerve-Tibial | Muscle-Skeletal | Whole-Blood | Adipose-Subcutaneous  Pancreas  Esophagus-Mucosa |
| **Protein coding** | Non-coding | Non-coding | Non-coding | Non-coding |
| **Variant type** | SNV | SNV | SNV | SNV |
| **Alleles** | G>A,C,T | A>G,T | G>A,C | G>A,T |
| **Chromosome** | 3:12232268 (GRCh38) | 5:75355259 (GRCh38) | 1:55086045 (GRCh38) | 7:44541277 (GRCh38) |
| **Functional Consequence** | Intergenic_variant | intron_variant | intron_variant,  genic_downstream_transcript_variant | 5_prime_UTR_variant,  upstream_transcript_variant,  genic_upstream_transcript_variant |
