# Supplementary material for: A Mendelian randomization study for drug repurposing reveals bezafibrate and fenofibric acid as potential osteoporosis treatments
Source: Front Pharmacol. 2023 Jul 20;14:1211302. doi: 10.3389/fphar.2023.1211302 (PMC10397407; doi:10.3389/fphar.2023.1211302)
Supplement: Supplementary file 3 [file Table3.docx]

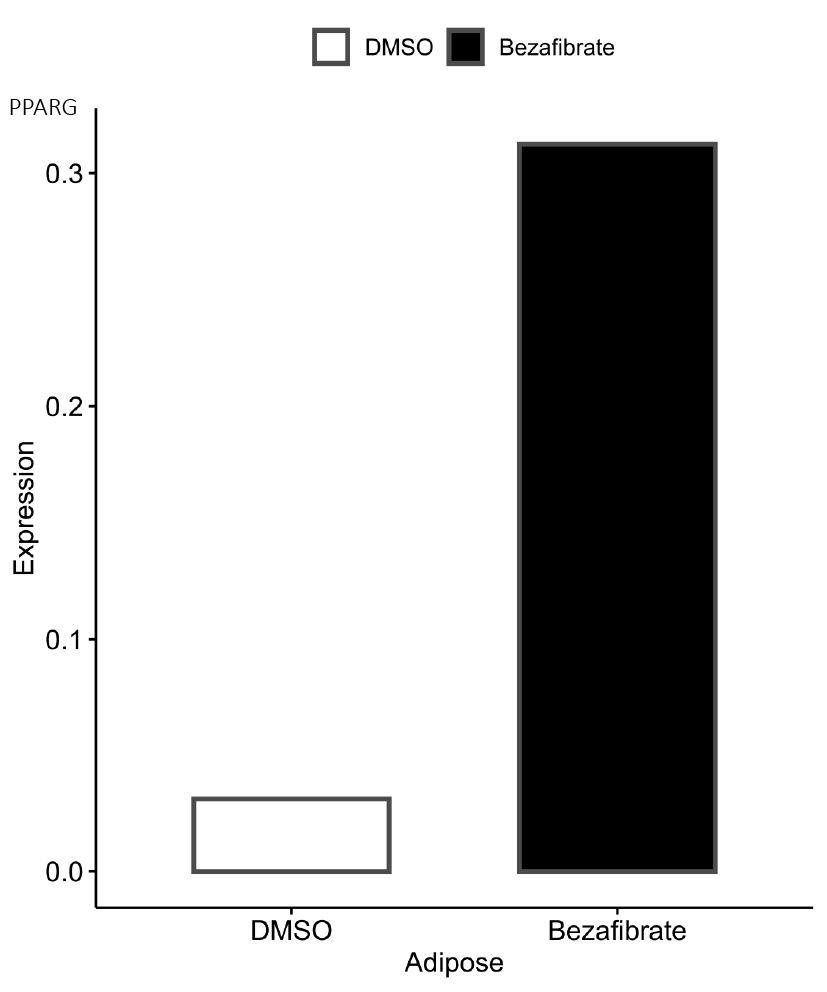
Supplementary Figure S1 *PPARG* expression level in adipose tissue by BZF-treatment in LINCS database

Note: DMSO, Dimethylsulfoxide
